# The Toxicity of Pneumonic Lungs

**Published:** 1918-11

**Authors:** 


***The Toxicity of Pneumonic Lungs.*** By CHARLES WEISS, JOHN A. KOLMER and EDWARD STEINFIELD. *The Journal of Infectious Diseases*, May, 1918.

In lobar pneumonia the toxemia is ascribed largely to toxic substances from virulent pneumococci.

While the toxic substance may be elaborated during lobar pneumonia insufficient amounts to account in large part for the toxic symptoms, in addition to probably influencing enzymic processes, our experiments show that its production *in vitro* is quite irregular, that large numbers of virulent pneumococci are required for its production, and that relatively large amounts of it are required to produce intoxication of animals. When it is remembered that sections of pneumonic lungs frequently show few pneumococci, it is questionable whether there is a sufficient amount of pneumotoxin produced in lobar pneumonia to account in whole for the toxemia of this disease.

With these considerations in mind, the authors have studied the exudate in lobar pneumonia as a further source of toxic substances. Riesman states that in pneumonia the bacterial toxemia has been overemphasized to the exclusion of everything else, whereas it is not improbable that some of the general symptoms are due to the exudate independently of the bacteria. The work of Rosenow and Arkin indicates that the exudate in lobar pneumonia is toxic, in that extracts of pneumonic lungs in salt solution, injected intravenously in dogs, produced blood pressure and respiratory disturbances slightly similar to those of protein and anaphylaxis in general.

*Toxicity of Extracts of Normal and Pneumonic Lungs* : Almost invariably the extracts of lungs secured soon after death, and in the stage of grave hepatization, contain pneumococci and other bacteria capable of killing animals in small doses.

The fatal toxic dose of pneumonic lung extract prepared by phenol sterilization is about 4 cc. per kilo. of weight when injected intraperitoneally in mice; the toxicity of fresh extract sterilized by thorough centrifuging and heating at 56 C. for 30 minutes is between 15 and 25 cc. per kilo. of weight.

By intravenous injection in guinea pigs, doses of phenolized extract greater than 0.5 cc. per kilo of weight are almost invariably fatal within a few minutes, producing convulsions similar to acute dyspnea anaphylactic intoxication.

Sterile extracts of pneumonic lungs in dogs, removed 48 hours after intrabronchial insufflation of virulent pneumococci, are more toxic than extracts of equal weights of normal dog lung.

The toxicity of extracts of normal and pneumonic lungs is decreased by heating, drying, and filtration through Berkefeld's filters.

Extracts of pneumonic lungs in grey hepatization are markedly hemolytic in larger lungs. Similar extracts of normal human lung tissue are found to have little or no hemolytic effect, and extracts of the exudates of lungs of dogs, removed 48 hours after the intrabronchial injection of virulent pneumococci and of sterile aleuronat, are generally non-hemolytic, indicating that appreciable amounts of hemolytic substance are not present in fresh pneumococcus exudate, but are present in human pneumonic lungs in grey hepatization and with early autolysis of the exudate.

The nature of the toxic and hemolytic substance or substances in extracts of pneumonic lungs in the later stages of pneumonia is unknown; it is probably allied with the protein fractions, and may be properly responsible for the production of the various systemic symptoms of lobar pneumonia ascribed to toxemia.

